# Non-contrast photon counting computed tomography of the head: optimized modeling of off-focal radiation to reduce calvarium-related tissue inhomogeneity

**DOI:** 10.1186/s12880-025-01718-w

**Published:** 2025-07-18

**Authors:** Arwed Elias Michael, Martin Petersilka, Denise Schoenbeck, Matthias Michael Woeltjen, Julius Henning Niehoff, Christoph Moenninghoff, Tanja Kurzendorfer, Jan Borggrefe, Lukas Goertz, Jan Robert Kroeger

**Affiliations:** 1 Department of Radiology, Neuroradiology and Nuclear Medicine, Johannes Wesling University Hospital, Ruhr University Bochum, Bochum, Germany; 2 Siemens Healthineers, Erlangen, Germany; 3 Department of Radiology and Neuroradiology, University Hospital of Cologne, Cologne, Germany; 4 Johannes Wesling University Hospital by Muehlenkreiskliniken AöR, Hans-Nolte-Straße 1, 32429 Minden, Germany

**Keywords:** Computed tomography, Photon counting computed tomography, Photon counting detector, Image quality, Non-contrast CT of the head, Calvarial artefacts, Off-focal radiation

## Abstract

**Background:**

Photon counting CT (PCCT) is a promising technique for neuroradiological CT examinations. In initial studies on non-contrast PCCT of the head (NCCT), however, artifacts close to the calvarium were noticed, which lead to an inhomogeneous representation of the brain tissue. In this study, a new software for image reconstruction to reduce artifacts is evaluated.

**Methods:**

In the new CT software developed by the manufacturer, off-focal radiation was remodeled and is mathematically corrected in the NCCT in data processing during image formation. For the evaluation, 60 patients with an NCCT in the currently used software and 44 patients in the new software were included retrospectively. A detailed quantitative analysis using multiple regions of interest and a qualitative analysis with a reading by experienced radiologists was performed to evaluate image quality and tissue homogeneity below the calvarium.

**Results:**

The new software reduced the inhomogeneity of the cortical brain tissue near the calvarium. As a quantitative measure, there is a clear reduction of the signal difference of the gray and white matter at different distances from the calvarium (*p* < 0.001). In the qualitative analysis, the inhomogeneity of the brain tissue was reduced, and the gray-white differentiation improved (*p* < 0.001) in the clinically used virtual monoenergetic image at 65 keV.

**Conclusions:**

Optimized modelling and mathematical correction of the off-focal radiation in the new software led to an effective reduction of the inhomogeneity of the cortical brain tissue and thus improved image quality.

## Introduction

Non-contrast cranial computed tomography (NCCT) is one of the most common CT examinations performed. Artifacts caused by the calvarium continue to be a problem even with the latest CT technologies [1, 2]. Photon counting CT (PCCT) is a new technique in routine clinical practice that offers many advantages in neuroradiologic imaging [3]. Because of the high number of NCCT, this examination played a role in early developmental steps in the field of PCCT [4]. Initial studies with preclinical PCCT have so far shown advantages in CT angiography of the head and neck [5]. For NCCT however, literature is sparse so far [6–9]. While NCCT in PCCT provided an overall good image quality in virtual monoenergetic images (VMI) in a recent study, remaining artifacts due to the calvarium were described, in which there are considerable attenuation differences of the brain matter depending on the distance to the calvarium [7]. The fact that the gray or white matter has very different CT values (Hounsfield units) in different locations can be called “inhomogeneity” of the tissue. In addition to established image quality parameters, this tissue homogeneity is also an important parameter in NCCT: In a high-quality CT image of the head, not pathologically altered gray and white matter should have the same CT values, regardless of the localization or distance to bony structures. With the currently clinically available PCCT, however, this tissue inhomogeneity substantially limits the clinical use of low-energy VMI and its inherent advantage of high gray to white matter contrast [7].

Different mechanisms can cause artifacts in the region of the skull. The best known of these are artifacts caused by hardening of the X-ray beam on dense structures, so called beam hardening artifacts [10]. Another cause is the influence of off-focal radiation [11]. Initiated by clinical experience at our institute, technical analyses by the manufacturer have shown that the influence of off-focal radiation is primarily responsible for the tissue inhomogeneity in the brain matter below the calvarium. Each CT device has complex software that is used for both data acquisition and data processing. Among other things, this software contains various algorithms for artifact reduction. As part of a major hardware and software update developed by the manufacturer for the currently clinically available PCCT (NAEOTOM Alpha, Siemens Healthineers, Erlangen, Germany), the off-focal radiation was remodeled and mathematically corrected in NCCT. The aim was to significantly reduce the inhomogeneity of the brain tissue below the calvarium. In the study presented here, the new software for image data processing and reconstruction for NCCT in PCCT will be evaluated. In addition to conventional parameters for image quality, the reduction of calvarium-related tissue inhomogeneity targeted in the development of the software will be investigated.

## Materials and methods

### Patient population

The study was conducted according to the guidelines of the Declaration of Helsinki and approved by the Institutional Ethics Committee of the Ruhr-Universität Bochum. Patients with examinations before and after the software update were included retrospectively. The last two weeks before and the first two weeks after the update were defined as the period for the inclusion of patients because a minimum number of 30 patients per group was expected within this period. Based on previous analyses, it was assumed that this group size would be adequate for the analysis. Informed consent was waived due to the retrospective study design. Each CT had been performed with a clinical protocol and with medical indication. Examinations with pronounced motion artifacts or artifacts due to foreign material were excluded. The data were anonymized.

### CT protocol and image acquisition

All CT scans were performed using the clinically approved photon-counting CT (NAEOTOM Alpha, Siemens Healthineers, Erlangen, Germany) with a spiral CT protocol. At first, patients were examined with the CT software version syngo CT VA50A (VA50A), after updating the software within a final validation and verification phase for the new software version the following patients were examined with the new software version syngo CT VB10 CUT (VB10) that incorporated a new algorithm to model extra-focal radiation. Due to the complex update, the raw data of the patients who were examined with the software VA50A could not be processed with the new software VB10. All patients were examined in supine position with moderate flexion in the cervical spine to perform axial acquisition in the orbitomeatal plane. Single collimation was 0.4 mm, total collimation 38.4 mm, and pitch factor was 0.55 with a rotation time of 0.5 s. Tube voltage was 120 kVp, and tube current was modulated due to the manufacturer’s program of dose modulation (image quality level 300). Organ-based dose modulation (X-CARE) was used to reduce the radiation applied to patients’ eyes. The matrix size was 512 × 512, the field of view (FOV) was optimized for the individual head size. The reconstruction kernel QR36 and the fourth level of iterative reconstruction (Quantum Iterative Reconstruction, QIR) were used for the spectral data sets. These were analyzed in the manufacturer-specific spectral workstation (syngo.via, VB80 CUT version, Siemens Healthineers, Erlangen, Germany). The images were reconstructed in axial view with a slice thickness of 3 mm and a slice increment of 3 mm.

### Software optimization

In contrast to an ideal, point-shaped focus, a real X-ray focus always has a certain spatial extension. Most of the radiation emanating from the anode results from a focused bundle of primary electrons reaching the anode with an spatial extension of a few 0.1 mm to 1 mm, which is optimized regarding the thermal load. In addition to focal spot size, image quality is also determined by the intensity distribution across the focus and by the so-called off-focal radiation (OFR). OFR refers to the proportion of X-ray radiation that originates from outside the desired focal spot: a certain proportion of the electrons that hit the anode in the primary beam are scattered back and accelerated to the anode a second time, thus generating lower-energy secondary X-ray radiation outside the original focal spot. This effect is counteracted by constructive methods in the design of the X-ray tube, but it cannot be fully eliminated.

In CT images, the effects of OFR are most prominent at the transition between soft tissue and bone, i.e. where attenuation values change abruptly. This is particularly the case in the region of the skull. One way to reduce the influence of the remaining OFR on the image quality is to model the residual OFR in the processing of the acquired data during image formation.

With syngo CT VB10, the modeling of OFR was improved over VA50, in particular for radiation components along the z-direction (i.e., the spatial direction along the patient axis).

**Fig. 1:**
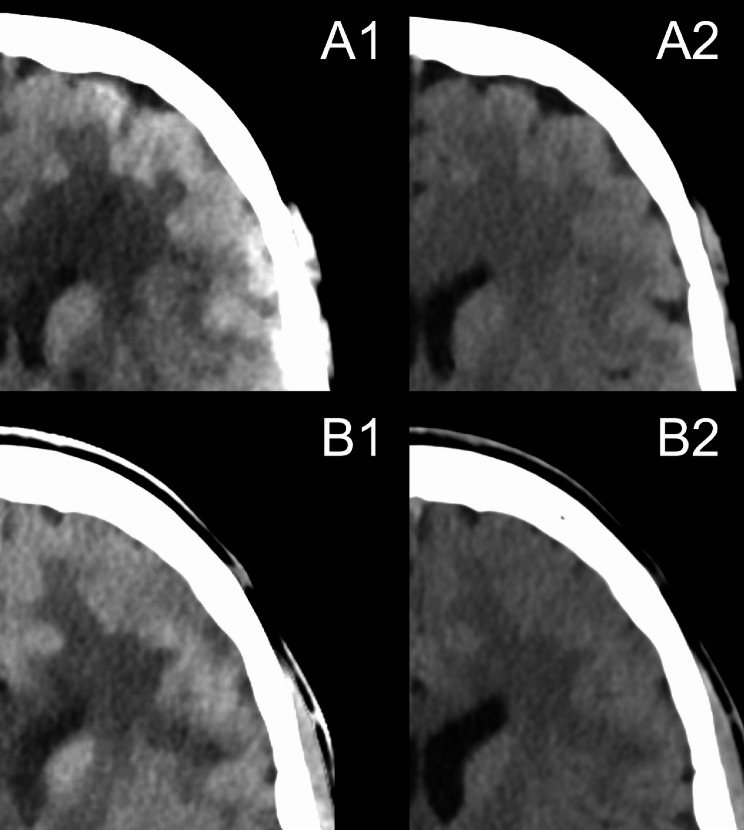
Inhomogeneity of the cortical brain tissue in axial CT images. The VMI 40 keV in VA50A (**A1**) and VMI 65 keV in VA50A (**A2**) as well as the VMI 40 keV in VB10 (**B1**) and VMI 65 keV in VB10 (**B2**) are shown. The representative section of the cortical brain tissue in VA50 shows the clear differences in attenuation with higher values close to the calvarium, which are particularly apparent in the VMI 40 keV. The brain tissue is displayed inhomogeneously. In VB10, this effect is significantly less pronounced and subjectively barely distinguishable even in VMI 40 keV. All images are displayed with equal window settings (C40/W80). VMI: virtual monoenergetic image; keV: kiloelectronvolt; C: window center; W: window width

This is why the VB10 should lead to improved image quality, particularly in the area of the calvarium (Fig. 1). Compared to (scintillation-based) EIDs, photon-counting detectors are more sensitive to lower-energy radiation, which is why OFR can be more pronounced in head imaging with PCCT. In this respect, optimizing the modeling and correction of OFR is of significance in PCCT.

### Quantitative image analysis

The quantitative analysis was performed by a trained medical student in collaboration with an experienced radiologist in training (5 years of experience with the evaluation of NCCT) and checked by a radiology consultant (7 years of experience). For the quantitative analysis, the procedure from a previous study [7] was adopted in precise detail, so 16 different ROIs were used. Of those, nine ROIs involved the gray and white matter of the supratentorial cortex at different distances to the calvarium (directly below and 5–10 mm). The next six ROIs involved deep gray and deep white matter. The last of those sixteen ROIs was placed in the pons between the petrous bones to provide a measure of artifacts analogous to the preliminary studies [6]. The average attenuation and its standard deviation were measured for all available virtual monoenergetic images from 40 keV to 190 keV for all ROIs. Parameters for assessing image quality were determined as described previously, in order to compare the study results [6, 12]. The signal of the ROIs was defined as the mean attenuation in Hounsfield units (HU). The signal difference between gray matter 5 mm and gray matter 20 mm below the calvarium was taken as a measure of tissue inhomogeneity. Noise was defined as the standard deviation (SD) of a ROI in HU. The signal-to-noise ratio (SNR) was defined as the quotient of the mean attenuation of the ROI (signal) and the SD of the ROI (noise). The contrast-to-noise ratio (CNR) was calculated as the quotient of the difference of the mean attenuation of two adjacent ROIs with gray matter (GM) and white matter (WM) and the square root of the sum of the variance of both ROIs [6].

### Qualitative image analysis

**Fig. 2:**
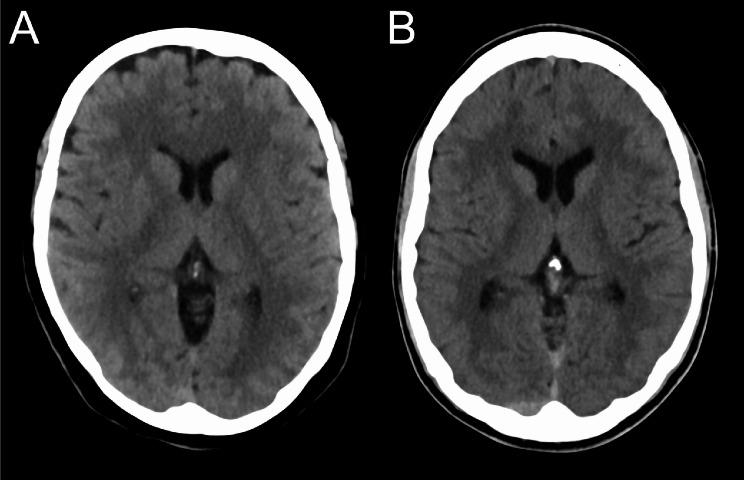
VMI 65 keV in VA50 and VB10 for axial CT images. **A**: VMI 65 keV in software VA50; **B**: VMI 65 keV in software VB10. The VMI 65 keV is currently the VMI used in clinical routine and was therefore selected for the qualitative analysis. In this VMI 65 keV, a closer look also reveals that in the VA50A the gray matter directly below the calvarium appears denser than cortical gray matter remote from the calvarium, for example, the cortical gray matter of the insula. Such a difference is subjectively hardly recognizable with the VB10.VMI: virtual monoenergetic image; keV: kilo electronvolt

The VMI 65 keV (Fig. 2) is currently used in our clinical routine and was therefore selected for a reading by four radiologists: two experienced neuroradiologists with 18 and 15 years of experience, two general radiologists with 9 and 7 years of experience. On a 5-point Likert scale, readers evaluated the homogeneity of gray and white matter below the calvarium in conjunction with the artifact burden (from 1 = “large signal differences within gray and white matter, many artifacts” to “homogeneous depiction within the gray and white matter, no artifacts”), and the gray white matter differentiation (from 1 = “severely limited gray-white differentiation, not diagnostic” to 5 = “optimal gray-white differentiation”). The readers were blinded to the software version used in reconstruction and postprocessing, every image was presented with equal standard windowing (C40/W80).

### Statistical analysis

The statistical software R [Version 4.1.0; 13] and RStudio (Version 2023.03.0 + 386) were used for data processing and analysis. The Shapiro–Wilk test was applied to test for normal distribution, the Levene test to check for homoscedasticity. A one-way ANOVA or the Friedman test were used to compare means of different ROIs or keV levels. P values were corrected using the Bonferroni method. For post hoc testing the paired T-test or the nonparametric Wilcoxon signed rank test were used.

For qualitative analysis, appropriate nonparametric procedures were used (Friedman test and Wilcoxon test). The interreader reliability was analyzed using the intraclass correlation coefficient (ICC; ICC(C,1), two-way mixed, single measures, consistency). If not stated otherwise, all data are presented as mean ± standard deviation.

## Results

### Patient population and radiation dose

Within the selected period 60 patients with an examination with VA50 and 44 patients with an examination VB10 were included. The mean age was 65.1 ± 19.4 years for VA50 and 69 ± 17.9 years for VB10. The effective tube current was 253.0 ± 21.5 mAs for VA50 and 258.0 ± 15.4 mAs for VB10. With regard to dose, the CTDIvol for VA50 was on average 45.7 ± 3.9 mGy (95% confidence interval: 44.7–46.7 mGy), for VB10 44.0 ± 2.7 mGy (95% confidence interval: 43.2–44.8 mGy). The dose length product was 737.0 ± 78.1 mGy*cm for VA50 and 751.0 ± 68.7 mGy*cm for VB10 (*p* = 0.35).

### Signal and tissue inhomogeneity

In the vast majority of all the 16 ROIs, the signal dropped from high values in the VMI at low keV to lower values at high keV in both VA50A and VB10. The only exceptions are the ROI in the white matter adjacent to the inferior basal ganglia and the ROI in the pons with VA50A. In these ROIs, the signal also increased with increasing keV in the VA50A, whereas there were no such exceptions in the VB10. In the critical region up to 20 mm below the calvarium, there were many significant differences in the signal of the gray and white matter between the software versions. In summary, in VB10 the signal in the VMI with lower keV was lower at 5 mm and 10 mm than in VA50A, at 15 mm and 20 mm it tended to be increased (*p* < 0.001, for the specific values of exemplary VMI see Table 1). At VMI with higher keV, the signal in VB10 was only slightly lower (*p* < 0.001, for specific values of exemplary VMI see Table 1).

**Table 1:** Signal in cortical Gray matter below the calvarium. The signal of the ROI in the Gray matter at the particular distance to the calvarium (5 mm to 20 mm) is shown in selected VMI (40 keV, 65 kev, 90 kev and 120 keV). GM: Gray matter; VMI: virtual monoenergetic image; keV: kilo electronvolt; sd: standard deviation; VA50A: software version VA50A; VB10: software version VB10

ROI	VMI	VA50A mean	VA50A sd	VB10 mean	VB10 sd	Corrected *p*
GM 5 mm	40 keV	68.56	10.06	56.69	5.65	< 0.001
GM 5 mm	65 keV	46.12	2.96	41.96	2.46	< 0.001
GM 5 mm	90 keV	38.63	2.03	36.58	1.84	< 0.001
GM 5 mm	120 keV	35.47	2.21	34.36	1.77	0.031
GM 10 mm	40 keV	59.42	7.44	57.08	5.67	0.689
GM 10 mm	65 keV	43.33	2.46	41.37	1.85	< 0.001
GM 10 mm	90 keV	37.55	1.74	35.65	1.55	< 0.001
GM 10 mm	120 keV	35.18	1.92	33.37	1.6	< 0.001
GM 15 mm	40 keV	53.02	6.4	54.43	4.71	1
GM 15 mm	65 keV	41.05	1.84	39.7	1.72	0.012
GM 15 mm	90 keV	36.71	1.48	34.44	1.33	< 0.001
GM 15 mm	120 keV	34.98	1.78	32.35	1.41	< 0.001
GM 20 mm	40 keV	48.79	6.51	50.7	4.5	0.780
GM 20 mm	65 keV	39.46	2.07	38.29	1.7	0.010
GM 20 mm	90 keV	35.94	1.82	33.41	1.16	< 0.001
GM 20 mm	120 keV	34.55	2.07	31.47	1.12	< 0.001

**Fig. 3:**
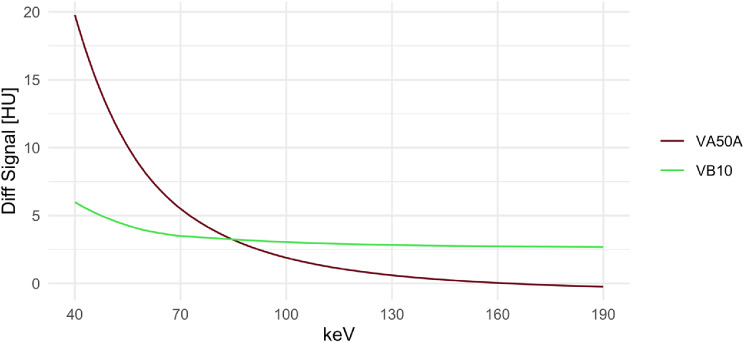
Inhomogeneity of Gray Matter Below the Calvarium. The signal difference of the gray matter at 5 mm and 20 mm below the calvarium is shown. This signal difference was taken as a measure of tissue inhomogeneity. With the VA50A, the difference in the VMI with lower keV was significantly higher than with the VB10. From 85 keV this changed and the signal difference tended to be slightly greater with VB10. Diff Signal: difference in signal / attenuation of gray matter at 5 mm an 20 mm below the calvarium; HU: Hounsfield units; keV: kilo electronvolt

These changes in signal below the calvarium resulted in the significantly reduced signal difference in VB10 in cortical gray and white matter 5 mm and 20 mm below the calvarium at low keV VMI as a measure for tissue inhomogeneity (Fig. 3). At VMI 40 keV in gray matter, for example, the signal difference was 19.77 ± 8.37 HU at VA50A, only 5.99 ± 5.62 HU at VB10 (corrected *p* < 0.001); at VMI 65 keV, 6.67 ± 3.19 HU at VA50A and only 3.67 ± 2.22 HU at VB10 (corrected *p* < 0.001). From 85 keV, the signal difference in gray matter in VB10 tended to be higher than in VA50A, at 90 keV this difference was not significant (corrected *p* = 1). For the VMI 120 keV, there was a difference of 0.91 ± 2.26 HU for VA50A and 2.89 ± 1.61 for VB10 (corrected *p* < 0.001).

### Noise

In the gray and white matter 5 mm and 10 mm below the calvarium, VB10 tended to show a slight reduction in noise across all keV levels compared to VA50A. This reduction was significant in the gray matter 5 mm below the calvarium at exemplary 40 keV (VA50A 6.6 ± 1.49 HU, VB10 5.23 ± 1.18 HU, corrected *p* < 0.001), in the other exemplarily selected VMI with 65 keV, 90 keV, and 120 keV there were no significant differences after Bonferroni correction.

In the deep gray matter and the adjacent white matter, the noise was practically identical in both software versions, for example in the thalamus in VMI 65 keV in VA50A 2.42 ± 0.46 HU, in VB10 2.44 ± 0.37 HU (corrected *p* = 1). Similarly, the noise did not differ in the ROI in the pons as a measure of the infratentorial artifacts (VMI 65 keV VA50A 3.39 ± 0.74 HU, VB10 3.52 ± 0.48 HU, corrected *p* = 0.631).

### SNR and CNR

For both cortical gray and white matter, the SNR was very similar in VA50A and VB10, with no significant differences for the selected VMI at 40 keV, 65 keV, 90 keV and 120 keV. Looking at tendencies, it should be noted that at higher keV, both cortically and in the deep gray and white matter, the SNR was lower in the VB10 than in the VA50A (for example, in the thalamus at 120 keV in VA50A 71.06 ± 24.01, in VB10 59.90 ± 17.60, corrected *p* = 0.293).

There were no significant differences in the CNR of cortical gray and white matter at 5 mm to 20 mm below the calvarium. There was a tendency for slightly higher values in VB10 (exemplary 10 mm below the calvarium at 65 keV in VA50A 1.12 ± 0.71, in VB10 1.37 ± 0.63, corrected *p* = 0.780). In the deep matter, this tendency reached significance in superior caudate nucleus (VMI 65 keV in VA50A 1.89 ± 0.70, in VB10 2.35 ± 0.46, corrected *p* = 0.006). In contrast, the tendency was reversed in the thalamus, where the CNR was slightly higher in the VA50A, but with no significant difference to VB10.

### Qualitative image analysis

**Fig. 4:**
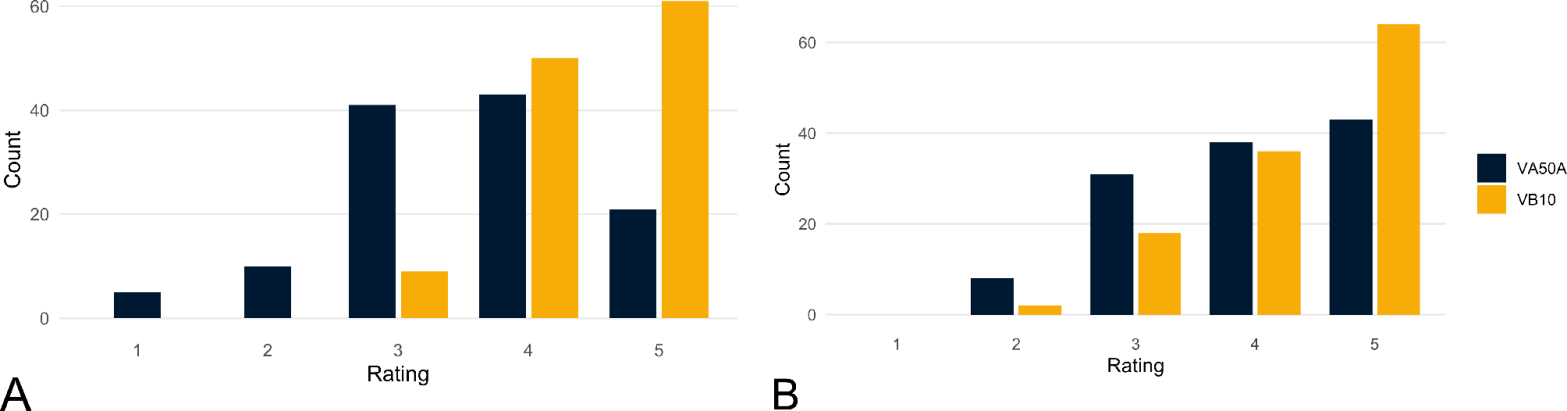
Rating of Tissue Homogeneity (**A**) and Gray-White Differentiation (**B**). Four radiologists rated the tissue homogeneity below the calvarium (from 1 = “large signal differences within gray und white matter, many artifacts” to “homogeneous depiction within the gray and white matter, no artifacts”) and gray-white differentiation (from 1 = “severely limited gray-white differentiation, not diagnostic” to 5 = “optimal gray-white differentiation”) on a 5-point Likert scale. VB10 significantly improves tissue homogeneity and gray-white differentiation

The currently clinically used VMI 65 keV was selected for the qualitative image analysis. Regarding tissue homogeneity, the VA50A images received an average rating of 3.54 ± 1.01 (first quartile (Q1) = 3, median = 4, third quartile (Q3) = 4), the VB10 images 4.43 ± 0.63 (Q1 = 4, median = 5, Q3 = 5). This difference was significant (corrected *p* < 0.001); interreader reliability was moderate (ICC = 0.488). The rating of gray-white differentiation was also in favor of the VB10. On average, the images of the VA50A were rated at 3.97 ± 0.94 (Q1 = 3, median = 4, Q3 = 5), while the VB10 was rated at 4.35 ± 0.80 (Q1 = 4, median = 5, Q3 = 5); this difference was also significant (corrected *p* = 0.002), interreader reliability was fair (ICC = 0.210). The exact distribution of the ratings is shown in Fig. 4.

## Discussion

In this study, the performance of a new CT software was evaluated for image data processing and reconstruction, particularly regarding the homogeneity of the visualization of the cortical brain tissue near the calvarium. It was shown that the new software version VB10 with optimized modeling and mathematical correction of OFR reduced the signal differences in gray and white matter depending on the distance to the calvarium in the clinically important VMI with low keV (up to 85 keV). Compared to the VA50A, the tissue inhomogeneity is thus significantly reduced, whereby this quantitative result was also clearly reflected in the qualitative reading. Noise was also significantly reduced by the VB10 in some VMI, with the majority of SNR and CNR showing no differences, or at most tendencies in favor of the VB10. Nevertheless, the gray-white differentiation was also rated significantly better in the reading.

Artifacts caused by the skull bone are a major challenge in NCCT. In particular, the posterior fossa with the adjacent dense bony structures has posed the greatest challenge due to such artifacts since the beginning of CT [14]. In studies on the image quality of NCCT, the so-called Posterior Fossa Artifact Index (PFAI) has therefore played a role very early on [15]. In more recent studies on the image quality of the NCCT in dual energy CT (DECT) and PCCT, this index was included [2, 6, 12]. The PFAI was also examined in this study but was not the primary object of software development and investigation. To take it up again briefly, there is no difference in the PFAI in the two software versions. In contrast to the artifacts of the posterior cranial fossa the artifacts adjacent to the calvarium have received too little attention so far. These artifacts are not only caused by beam hardening, but in particular by the influence of OFR. They lead to the described signal differences in the same type of brain tissue and thus to an inhomogeneous depiction of tissue and deteriorated image quality. This inhomogeneity of gray and white matter in low keV VMI is also evident in images from studies using advanced DECT [2, 12]; thus, it is not a phenomenon that is new with PCCT [7]. However, due to the significantly increased sensitivity of the photon counting detector to low energy photons compared to EID, the OFR has a greater influence on the PCCT [16]. Optimized modeling and mathematical correction of OFR can improve image quality in modern CT techniques [11]. In this study, the brain tissue inhomogeneity in NCCT could be significantly reduced as intended with the help of such optimized modelling. No literature exists to date on the effect of this optimization in photon counting NCCT, which is why a detailed assessment and further discussion is difficult here.

Since the introduction of DECT, it has been possible to generate VMI [17], which make it possible to reduce beam hardening artifacts in many areas [18]. This benefit has also been demonstrated for CT of the head [19]. The technique of photon counting CT also promises further advantages regarding the reduction of artifacts; analogous to DECT, the reduction of beam hardening artifacts has been demonstrated by using VMI [20]. About NCCT in PCCT, the study situation is sparse to date, also regarding the assessment of artifacts. In addition to beam hardening artifacts, artifacts caused by OFR also pose a problem in calculation of VMI, particularly in the low energy spectrum. Previous studies have clearly shown that the image quality of VMI with low keV is limited in PCCT [6, 7]. At the same time, however, the better soft tissue contrast in the low energy VMI should provide diagnostic advantages, which is why optimizing the image quality could be crucial here. The aim was to improve this image quality so that the low keV VMI can be reevaluated in the future and can be used clinically with their strengths regarding tissue contrast. The optimization of the software presented here is an important step in this development. The question in older studies as to which VMI is currently most suitable for clinical work could require re-evaluation.

This study has several limitations. There is the retrospective study design and the fact that it was not possible to examine the same patients twice at short intervals with the different versions. Unfortunately, it was also not possible to further process the raw data of a study with both software versions. Although the evidence of improved image quality is clear, it has not been evaluated whether this translates to a better detection of intracranial pathologies such as stroke hypodensities in ASPECTS reading.

In summary, this study demonstrated a significant improvement in image quality of NCCT in PCCT with a new software version containing a new algorithm for modeling and mathematical correction of the OFR, by reducing the inhomogeneity of the cortical brain tissue adjacent to the calvarium. This software has been developed by the manufacturer for the only clinically available PCCT system (NAEOTOM Alpha, Siemens Healthineers, Erlangen) and will soon be introduced into clinical routine by the manufacturer due to its convincing performance.

## Data Availability

The data are available on reasonable request.
